# Unveiling Key Genes and Unique Transcription Factors Involved in Secondary Cell Wall Formation in *Pinus taeda*

**DOI:** 10.3390/ijms252111805

**Published:** 2024-11-03

**Authors:** Wei Ding, Zhonghua Tu, Bin Gong, Zhaolei Deng, Qian Liu, Zhenjun Gu, Chunxia Yang

**Affiliations:** 1 Jiangxi Academy of Forestry, Nanchang 330013, China; njfu_ding@126.com (W.D.); zhonghuatu@jxlky.cn (Z.T.); g18070090393@163.com (B.G.); dengzhaolei97@126.com (Z.D.); lq_lky18670223641@126.com (Q.L.); 2 Jiangxi Provincial Key Laboratory of Improved Variety Breeding and Efficient Utilization of Native Tree Species, Nanchang 330013, China

**Keywords:** *Pinus taeda*, secondary cell wall formation, lignin, cellulose, and hemicellulose biosynthesis, NAC transcription factor, MYB transcription factor

## Abstract

*Pinus taeda* is a key timber species, and extensive research has been conducted on its wood formation. However, a comprehensive investigation into the biosynthetic pathways of lignin, cellulose, and hemicellulose in *P. taeda* is lacking, resulting in an incomplete understanding of secondary cell wall (SCW) formation in this species. In this study, we systematically analyzed transcriptomic data from previously published sources and constructed detailed pathways for lignin, cellulose, and hemicellulose biosynthesis. We identified 188 lignin-related genes and 78 genes associated with cellulose and hemicellulose biosynthesis. An RT-qPCR highlighted 15 key lignin biosynthesis genes and 13 crucial genes for cellulose and hemicellulose biosynthesis. A STEM analysis showed that most essential enzyme-coding genes clustered into Profile 14, suggesting their significant role in SCW formation. Additionally, we identified seven NAC and six MYB transcription factors (TFs) from atypical evolutionary clades, with distinct expression patterns from those of the previously characterized *NAC* and *MYB* genes, indicating potentially unique functions in SCW formation. This research provides the first comprehensive overview of lignin, cellulose, and hemicellulose biosynthetic genes in *P. taeda* and underscores the importance of non-canonical NAC and MYB TFs, laying a genetic foundation for future studies on SCW regulatory mechanisms.

## 1. Introduction

Wood is a vital biomass resource for producing timber, paper, chemicals, and biofuels. The process of wood formation, known as xylogenesis, involves several stages: cell division, cambial initial expansion, xylem cell differentiation, secondary cell wall (SCW) deposition, and programmed cell death (PCD) [1,2,3]. Typically, SCWs are enriched with lignin, cellulose, and hemicellulose [2]. Lignin, a key component of SCW, plays a crucial role in facilitating water and mineral transport and providing mechanical strength [4]. Lignin consists of three primary units: *ρ*-hydroxyphenyl (H), syringyl (S), and guaiacyl (G) [2]. There are significant differences in the composition and quantity of lignin between gymnosperms and angiosperms. Gymnosperm wood contains 25% to 35% lignin, predominantly of the HG type, with G units being the most abundant and H units present in minor amounts [5,6,7,8]. In contrast, angiosperm wood contains 15% to 28% lignin, primarily of the GS type, with the proportion of S units varying between species [5,6,7].

Lignin monomer biosynthesis begins with the phenylpropanoid pathway [1,8,9]. Through the action of several enzymes, such as phenylalanine ammonia-lyase (PAL), cinnamate 4-hydroxylase (C4H), 4-coumarate-CoA ligase (4CL), *ρ*-coumarate 3-hydroxylase (C3H), caffeoyl shikimate esterase (CSE), caffeic acid 3-O-methyltransferase (COMT), ferulate-5-hydroxylase (F5H), shikimate *O*-hydroxycinnamoyltransferase (HCT), caffeoyl-CoA *O*-methyltransferase (CCoA-OMT), cinnamoyl-CoA reductase (CCR), and cinnamyl-alcohol dehydrogenase (CAD), phenylalanine is converted into the three lignin precursors: *ρ*-coumaryl alcohol, coniferyl alcohol, and sinapyl alcohol [5,10,11]. These precursors are then polymerized into H, G, and S lignin via the catalytic actions of peroxidase (POX) and laccase (LAC) enzymes [12].

Cellulose, another critical SCW component, is synthesized through a distinct pathway. Cellulose synthesis begins with UDP-glucose, catalyzed by cellulose synthases (CESAs) [13,14]. UDP-glucose is derived from two main pathways: one involves sucrose cleavage by sucrose synthase (SUS), and the other involves glucose-1-phosphate catalysis by UDP-Glc pyrophosphorylase (UGP) [15,16]. Hemicellulose, an essential component of plant cell walls, consists of heteropolysaccharides composed of various monosaccharides linked in diverse arrangements [17,18]. Hemicellulose structures include xylan, xyloglucan, mannan, and β-(1→3, 1→4)-glucan. Xylan and mannan are the predominant hemicellulose types in grasses, hardwoods, and softwoods [17,18]. In *Arabidopsis thaliana*, xylan synthesis begins with UDP-glucose, which is converted into UDP-D-xylose via UDP-glucose dehydrogenase (UGD) and UDP-xylose synthase (UXS) [10]. The irregular xylem (IRX) enzymes—IRX10 (IRX10L), IRX9L, IRX7, and IRX14—then catalyze the synthesis of 1,4-β-D-xylan from UDP-D-xylose [19,20]. The xylan polymer is further modified by the glucuronic acid substitution of xylan (GUX) and reduced wall acetylation (RWA), leading to the formation of functional xylan [21,22,23,24].

SCW formation is a highly complex process, regulated by numerous transcription factors (TFs). Among these, NAC and MYB TFs are recognized as master regulators [1,25,26]. Vascular-related NAC domain (VND), NAC secondary wall thickening promoting factor (NST), secondary wall-associated NAC domain protein (SND), and SOMBRERO (SMB) subfamily TFs play critical roles in SCW formation [26,27]. Collectively referred to as VND/NST/SMB (VNS) or wood-associated NAC domain (WND) TFs, these first-layer regulators control SCW formation by activating secondary regulatory networks. For example, *A. thaliana* AtNST1/2, AtSND1, AtVND1/2/3/4/5/6/7, *Populus trichocarpa* PtrSND2/3/4/5, and *P. deltoides* PdWND3A have all been shown to play pivotal roles in SCW formation [28,29,30,31,32,33]. VNS TFs regulate second-layer master regulators such as AtMYB46 and AtMYB63, which subsequently control other MYB TFs (e.g., AtMYB4/6/7/20/32/42/43/52/54/58/63/69/85/99/103) and additional TFs (e.g., KNAT7), ultimately governing the expression of SCW-related genes [25,26,34].

Loblolly pine (*Pinus taeda*), native to the southeastern United States, is the second most abundant tree species in the country [35,36]. Its adaptability, high wood and fiber yield, and rapid growth make it a valuable species for timber, paper, and resin production, leading to its introduction in regions such as Brazil and China [36]. Studies on lignin biosynthesis in *P. taeda* have identified NAC TFs such as PtaVNS1/2/3/5, which promote SCW deposition by regulating genes like CESA, XYLEM CYSTEINE PEPTIDASE 1 (XCP1), and PtaMYB4 [37]. MYB TFs, including PtaMYB1, PtaMYB4, and PtaMYB8, positively regulate monolignol biosynthesis, while PtaMYB14 and PtaMYB15, which possess the ERF-associated amphiphilic repression (EAR) motif, act as the negative regulators of lignin biosynthesis [9,38,39,40,41,42,43]. However, these NAC and MYB TFs represent only a fraction of the complex regulatory network involved in SCW formation in *P. taeda*. It is noteworthy that most previous studies have focused on lignification in the aerial parts of the tree, with limited research on root lignification. Furthermore, there has been no comprehensive characterization of the genes involved in the biosynthesis of lignin, cellulose, and hemicellulose in *P. taeda*.

To address these gaps, we utilized transcriptomic data from *P. taeda* bark, buds, needles, roots, and twigs obtained from public databases, and applied homology-based alignment, reverse transcription-quantitative polymerase chain reaction (RT-qPCR), and short time-series expression miner (STEM) analyses [44]. We identified 188 genes involved in lignin biosynthesis and 78 genes associated with the synthesis of cellulose and hemicellulose, including 28 key enzyme-coding genes. These enzyme genes were predominantly clustered into Profile 14, underscoring the importance of these genes in SCW formation. Additionally, we discovered seven NAC TFs outside of the VNS family and six MYB TFs that are active in different tissues compared to the previously identified *NAC* and *MYB* genes regulating SCW formation in *P. taeda*. This research enhances our understanding of the biosynthetic pathways for lignin, cellulose, and hemicellulose in *P. taeda*, expands the scope of research on NAC and MYB TFs involved in SCW regulation, and establishes a genetic foundation for future investigations into the regulatory mechanisms of SCW formation in *P. taeda*.

## 2. Results

### 2.1. Overview of RNA Sequencing

Using the loblolly pine genome as a reference, the RNA sequencing of bark, bud, needle, root, and twig samples identified 116,705 transcripts from 67,790 genes [44]. The majority of the genes (69.30%) produced only one transcript, while 30.70% generated multiple transcripts (two or more) (Figure S1A). The gene expression levels were quantified using FPKM values, revealing a large number of genes with low expression (FPKM < 1) across all the tissues (Figure S1B). After filtering out these low-expression genes (FPKM < 1 in all tissues), 40,639 genes remained (Figure S1C).

### 2.2. Identification of Lignin Biosynthesis-Related Genes and Potential Key Genes

**Figure 1:**
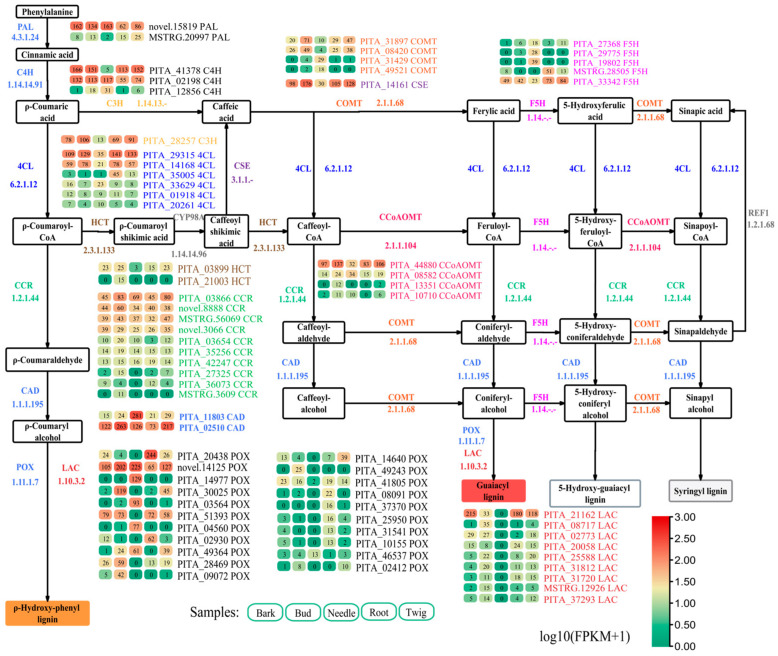
Crucial genes (FPKM values ≥ 10) in the lignin biosynthesis pathway. The log10(FPKM+1) values of genes were used to generate the heatmap of the gene expression levels. The numerical values in the heatmap represent the FPKM values of genes. PAL, phenylalanine ammonia-lyase; C4H, cinnamate 4-hydroxylase; 4CL, 4-coumarate-CoA ligase; CCR, cinnamoyl-CoA reductase; CAD, cinnamyl-alcohol dehydrogenase; POX, peroxidase; LAC, laccase; C3H, p-coumarate 3-hydroxylase; HCT, shikimate *O*-hydroxycinnamoyltransferase; CSE, caffeoyl shikimate esterase; COMT, caffeic acid 3-*O*-methyltransferase; CCoAOMT, caffeoyl-CoA *O*-methyltransferase; F5H, ferulate-5-hydroxylase.

A homology-based approach identified 188 lignin biosynthesis-related genes from the 40,639 filtered genes (Figure S2). From these, 70 genes with FPKM values exceeding 10 were selected, including 2 *PAL* genes, 3 *C4H* genes, 1 *C3H* gene, 6 *4CL* genes, 2 *HCT* genes, 1 *CSE* gene, 10 *CCR* genes, 2 *CAD* genes, 4 *COMT* genes, 4 *CCoAOMT* genes, 5 *F5H* genes, 21 *POX* genes, and 9 *LAC* genes (Figure 1). A heatmap analysis indicated that several of these 70 genes were highly expressed in the bark, root, and twig, while exhibiting low expression in needles, suggesting their potential role as key lignin biosynthesis genes. Notable examples include PITA_41378 (*C4H*), PITA_28257 (*C3H*), PITA_31897 (*COMT*), PITA_44880 (*CCoAOMT*), PITA_20438 (*POX*), PITA_30025 (*POX*), and PITA_21162 (*LAC*) (Figure 1).

**Figure 2:**
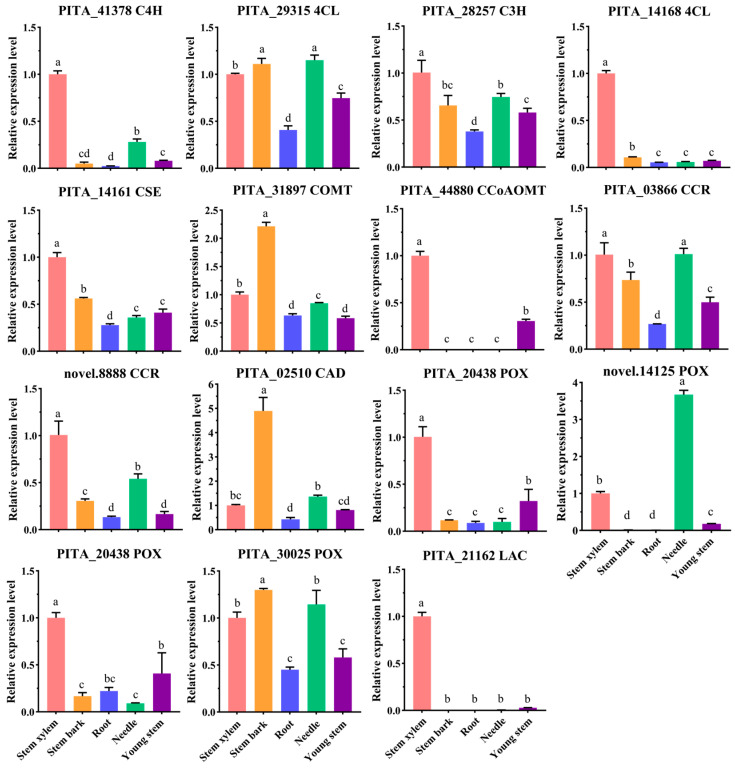
RT-qPCR analysis of lignin biosynthesis-related genes in the five tissues. The salmon, orange, royal blue, spring green, and purple bars represent the relative expression levels of genes in the stem xylem, stem bark, root, needle, and young stem (unlignified), respectively. The error bars represent the SDs from three replicates. The Duncan test was used to determine the significance of gene expression differences between the different tissues. The different lowercase letters indicate significant differences in the gene expression levels between the different tissues (*p* < 0.05).

Fifteen highly expressed genes were selected for further expression analysis in the stem xylem, stem bark, root, needle, and young (unlignified) stem. The RT-qPCR results revealed that PITA_14168 (*4CL*) and PITA_21162 (*LAC*) exhibited strong tissue-specific expression in stem bark (Figure 2). In contrast, PITA_44880 (*CCoAOMT*) and PITA_24038 (*POX*) were specifically expressed in the stem xylem and young stem (Figure 2). PITA_31897 (*COMT*) and PITA_02510 (*CAD*) showed high expression in the stem bark (Figure 2), whereas PITA_41378 (*C4H*), PITA_14161 (*CSE*), novel.8888 (*CCR*), and PITA_20438 (*POX*) were highly expressed in the stem xylem (Figure 2). The genes PITA_29315 (*4CL*) and PITA_30025 (*POX*) exhibited similar expression patterns, being highly expressed in the stem xylem, stem bark, and needle but lower in the root (Figure 2). Consistent with RNA-seq data, RT-qPCR indicated that novel.14125 (*POX*) had the highest expression in needles, followed by the stem xylem (Figure 2). Collectively, these findings suggest that PITA_41378 (*C4H*), PITA_14161 (*CSE*), novel.8888 (*CCR*), PITA_20438 (*POX*), PITA_31897 (*COMT*), PITA_02510 (*CAD*), PITA_44880 (*CCoAOMT*), PITA_24038 (*POX*), PITA_14168 (*4CL*), and PITA_21162 (*LAC*) play key roles in lignin biosynthesis in loblolly pine.

### 2.3. Overview of the Cellulose and Hemicellulose Biosynthesis Pathways and Key Gene Identification

**Figure 3:**
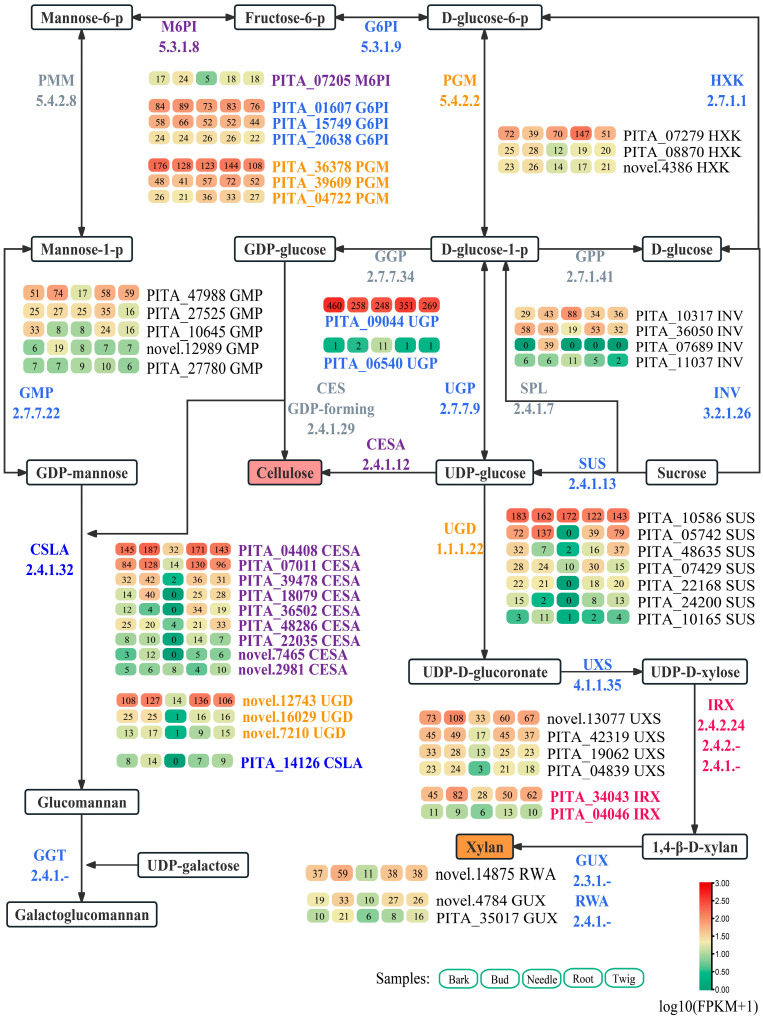
Main genes (FPKM values ≥ 10) in the cellulose and hemicellulose biosynthesis pathways. The log10(FPKM+1) values of genes were used to generate the heatmap of the gene expression levels. The numerical values in the heatmap represent the FPKM values of genes. CESA, cellulose synthase; SUS, sucrose synthase; SPL, sucrose phosphorylase; UGP, UDP-Glc pyrophosphorylase; UGD, UDP-glucose dehydrogenase; UXS, UDP-xylose synthase; IRX, irregular xylem; GUX, glucuronic acid substitution of xylan; RWA, reduced wall acetylation; GPP, glucose-1-phosphate phosphodismutase; INV, invertase; HXK, hexokinase; PGM, phosphoglucomutase; G6PI, glucose-6-phosphate isomerase; M6PI, mannose-6-phosphate isomerase; PMM, phosphomannomutase; GMP, GDP-D-mannose pyrophosphorylase; GGP, GDP-D-glucose pyrophosphorylase; CSLA, cellulose synthase-like A; GGT, glucomannan-1,6-galactosyltransferase.

In reference to previous studies, 78 genes associated with cellulose and hemicellulose biosynthesis were identified from the 40,639 filtered genes through homology searches (Figure S3). From these, 50 genes with FPKM values greater than 10 were selected, including 9 *CESA* genes, 3 *UGD* genes, 4 *UXS* genes, 2 *IRX* genes, 2 *GUX* genes, 1 *RWA* gene, 7 *SUS* genes, 4 invertase (*INV*) genes, 3 hexokinase (*HXK*) genes, 2 *UGP* genes, 3 glucose-6-phosphate isomerase (*G6PI*) genes, 1 mannose-6-phosphate isomerase (*M6PI*) gene, 3 phosphoglucomutase (*PGM*) genes, 5 GDP-D-mannose pyrophosphorylase (*GMP*) genes, and 1 cellulose synthase-like A (*CSLA*) gene (Figure 3). Similar to lignin biosynthesis, several highly expressed genes in these pathways exhibited lower expression levels in needles compared to the bark, root, and twig, such as PITA_04408 (*CESA*), PITA_07011 (*CESA*), PITA_05742 (*SUS*), PITA_42319 (*UXS*), novel.12743 (*UGD*), and PITA_34043 (*IRX*) (Figure 3).

**Figure 4:**
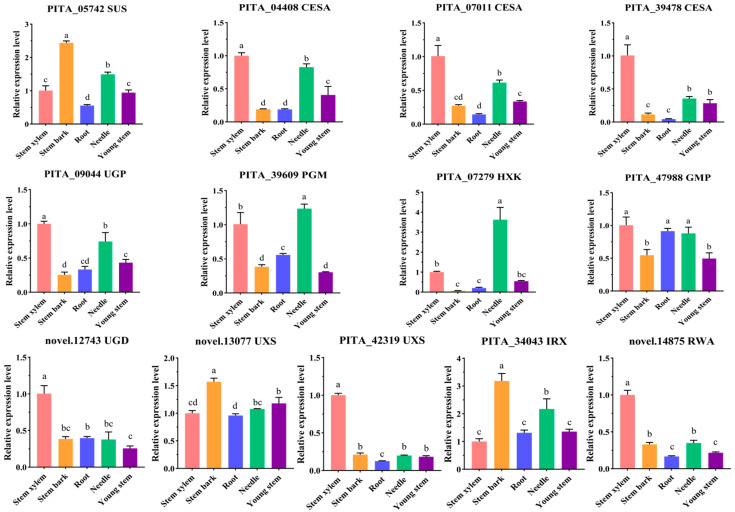
RT-qPCR analysis of the genes related to cellulose and hemicellulose biosynthesis in the five tissues. The salmon, orange, royal blue, spring green, and purple bars represent the relative expression levels of genes in the stem xylem, stem bark, root, needle, and young stem (unlignified), respectively. The error bars represent the SDs from three replicates. The Duncan test was used to determine the significance of gene expression differences between the different tissues. The different lowercase letters indicate significant differences in the gene expression levels between the different tissues (*p* < 0.05).

Thirteen highly expressed genes related to cellulose and hemicellulose biosynthesis were selected for RT-qPCR analysis to examine their expression levels in the stem xylem, stem bark, needle, root, and young stem. The RT-qPCR results showed that PITA_04408 (*CESA*), PITA_07011 (*CESA*), PITA_39478 (*CESA*), PITA_09044 (*UGP*), novel.12743 (*UGD*), PITA_42319 (*UXS*), novel.14875 (*RWA*), and PITA_47988 (*GMP*) were most highly expressed in the stem xylem and also showed significant expression in the needle and young stem (Figure 3). In contrast, PITA_39609 (*PGM*) and PITA_07279 (*HXK*) had the highest expression in needles (Figure 4). These findings suggested that genes such as PITA_04408 (*CESA*), PITA_07011 (*CESA*), PITA_39478 (*CESA*), PITA_09044 (*UGP*), novel.12743 (*UGD*), PITA_42319 (*UXS*), novel.14875 (*RWA*), PITA_47988 (*GMP*), PITA_05742 (*SUS*), novel.13077 (*UXS*), and PITA_34043 (*IRX*) were likely involved in cellulose, hemicellulose, and galactoglucomannan biosynthesis in loblolly pine.

### 2.4. Prediction of TFs and Validation of Expression Patterns

**Figure 5:**
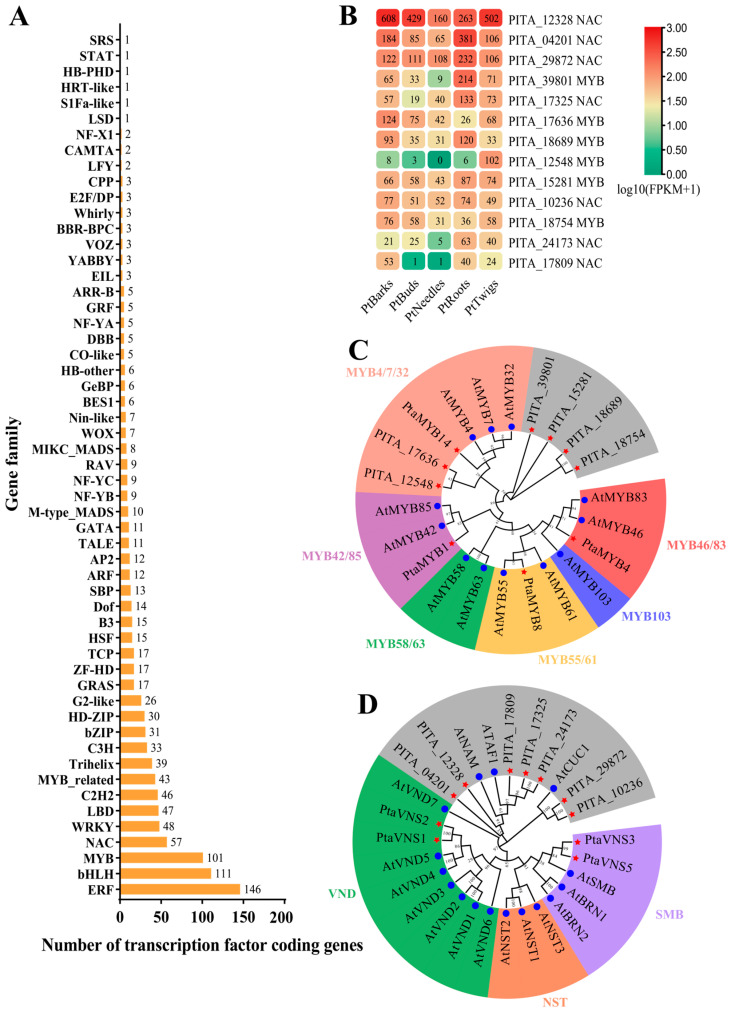
Statistics of transcription factors and the analysis of highly expressed MYB and NAC genes. (**A**) The statistics of transcription factor families in the RNA sequencing data. (**B**) The heatmap of highly expressed NAC and MYB genes (FPKM values > 50). (**C**,**D**) The phylogenetic trees of MYB and NAC proteins from *P. taeda* (red star) and *A. thaliana* (blue circle). The Neighbor-Joining method was used to construct the phylogenetic trees with the Jones–Taylor–Thornton model and 1000 bootstrap replications. The MYB protein phylogenetic tree includes 12 *A. thaliana* MYB proteins and 10 *P. taeda* MYB proteins, which were clustered into seven groups. The NAC protein phylogenetic tree includes 16 *A. thaliana* NAC proteins and 11 *P. taeda* NAC proteins, which were clustered into four groups.

TFs are critical regulators of SCW formation. From the 40,639 detected genes, we identified 1047 TF-encoding genes, with the *ERF* family being the most abundant, followed by the *bHLH*, *MYB*, *NAC*, and *WRKY* families. Previous studies have highlighted the roles of *PtaMYB1* (PITA_47805), *PtaMYB4* (PITA_05699), *PtaMYB8* (PITA_06912), *PtaMYB14* (PITA_48233), *PtaVNS1* (PITA_19394), *PtaVNS2* (PITA_33113), *PtaVNS3* (PITA_06801), and *PtaVNS5* (PITA_38627) in SCW formation in loblolly pine [37,38,39,40]. However, RNA-seq data indicated low expression levels of these eight genes in the bark, root, and twig samples (Figure S4). Specifically, *PtaVNS1* (PITA_19394) and *PtaVNS2* (PITA_33113) were excluded due to very low expression (FPKM < 1) (Figure S4). Interestingly, seven *NAC* and six *MYB* genes showed high expression levels in the bark, root, and twig samples (Figure 5B).

To better understand the relationship between these highly expressed *MYB* and *NAC* genes and the previously reported *PtaMYB* and *PtaNAC* genes, we constructed phylogenetic trees for MYB and NAC proteins. The analysis revealed that PtaMYB1 (PITA_47805), PtaMYB4 (PITA_05699), PtaMYB8 (PITA_06912), and PtaMYB14 (PITA_48233) were classified into the MYB42/85, MYB46/83, MYB55/61, and MYB4/7/32 clades, respectively (Figure 5C). Among the six newly identified MYB TFs, only PITA_17636 and PITA_12548 clustered into the MYB4/7/32 clade, while PITA_39801, PITA_15281, PITA_18689, and PITA_18754 grouped into unknown clades. This suggests the involvement of additional MYB TFs outside the traditional clades in SCW synthesis in loblolly pine.

The VNS family, known for its role in SCW synthesis regulation, was divided into VND, NST, and SMB clades, which aligned with our phylogenetic tree results (Figure 5D) [37]. The previously reported PtaVNS1 (PITA_19394) and PtaVNS2 (PITA_33113) were classified into the VND clade, while PtaVNS3 (PITA_06801) and PtaVNS5 (PITA_38627) were grouped into the SMB clade (Figure 5D). The seven newly identified NAC TFs were assigned to non-VNS clades, including PITA_04201, PITA_12328, PITA_17809, PITA_17325, PITA_24173, PITA_29872, and PITA_10236 (Figure 5D). Research indicates that non-VNS NAC TFs can also regulate SCW synthesis, suggesting that these NAC TFs might play roles in SCW formation [4].

**Figure 6:**
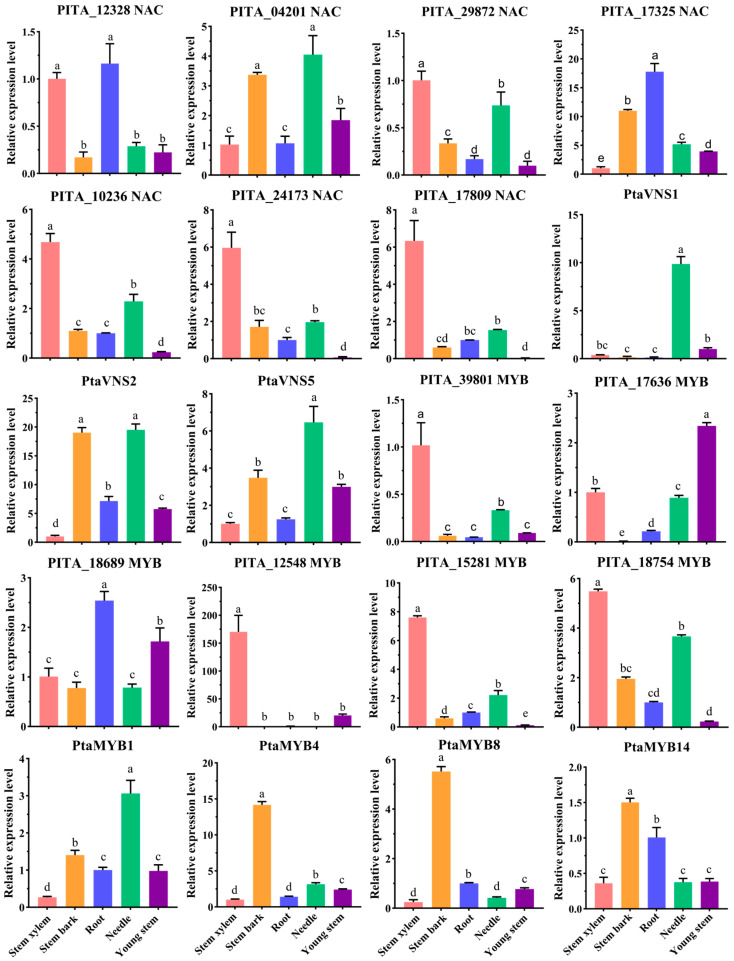
RT-qPCR analysis of *MYB* and *NAC* genes in the five tissues. The salmon, orange, royal blue, spring green, and purple bars represent the relative expression levels of genes in the stem xylem, stem bark, root, needle, and young stem (unlignified), respectively. The error bars represent the SDs from three replicates. The Duncan test was used to determine the significance of gene expression differences between the different tissues. The different lowercase letters indicate significant differences in the gene expression levels between the different tissues (*p* < 0.05).

To further verify the functions of these NAC and MYB genes, we analyzed their expression patterns in the stem xylem, stem bark, root, needle, and young stem. Among the four reported *PtaVNS* genes, only *PtaVNS3* was undetected, whereas *PtaVNS1*, *PtaVNS2*, and *PtaVNS5* exhibited high expression levels in the needles (Figure 6). Additionally, *PtaVNS2* and *PtaVNS5* showed elevated expression levels in the stem bark (Figure 6). Notably, PITA_04201 (*NAC*) demonstrated an expression pattern similar to *PtaVNS5*, suggesting a potential functional similarity (Figure 6). PITA_10236 (*NAC*), PITA_24173 (*NAC*), PITA_17809 (*NAC*), and PITA_29872 (*NAC*) were predominantly expressed in the stem xylem and needles (Figure 6). PITA_12328 (*NAC*) had significantly higher expression levels in the root and stem xylem, while PITA_17325 (*NAC*) was highly expressed in both the root and stem bark (Figure 6). Interestingly, *PtaMYB1* shared an expression pattern with *PtaVNS5* and PITA_04201 (*NAC*). Furthermore, *PtaMYB4*, *PtaMYB8*, and *PtaMYB14* displayed the highest expression levels in the stem bark, with lower levels in the needle, stem xylem, and young stem (Figure 6). PITA_39801 (*MYB*), PITA_15281 (*MYB*), and PITA_18754 (*MYB*) were predominantly expressed in the stem xylem but showed lower expression levels in the young stem, whereas PITA_17636 (*MYB*) had the highest expression level in the young stem (Figure 6). PITA_12548 (*MYB*), belonging to the same clade as PITA_17636, exhibited strong tissue specificity in the stem xylem (Figure 6). PITA_18689 (*MYB*) was most highly expressed in the root, followed by the young stem, with significantly lower levels in the stem xylem, stem bark, and needle (Figure 6). These findings suggest that the highly expressed *MYB* and *NAC* genes identified in this study may influence SCW formation in various tissues or developmental stages.

### 2.5. STEM Analysis of Selected Genes

**Figure 7:**
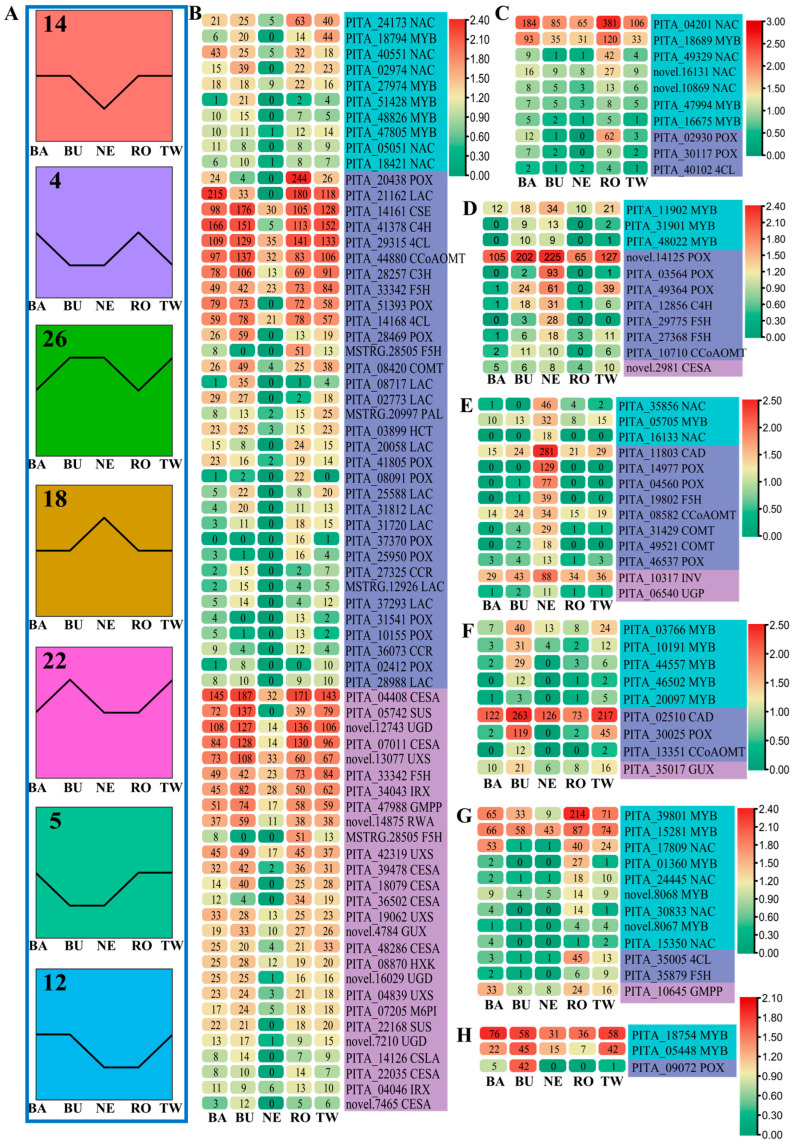
STEM analysis of the filtered genes. BA, barks; BU, buds; NE, needles; RO, roots; TW, twigs. (**A**) Seven significantly clustered profiles. (**B**–**H**) The heatmaps of NAC, MYB, lignin biosynthesis-related genes, and cellulose, and hemicellulose biosynthesis-related genes in Profile 14, Profile 4, Profile 26, Profile 18, Profile 22, Profile 5, and Profile 12, respectively. The log10(FPKM+1) values of genes were used to generate the heatmaps of gene expression levels. The numerical values in the heatmaps represent the FPKM values of genes. Only the genes with FPKM values greater than 10 are displayed in the figure.

Further clustering of the highly expressed enzyme-coding genes and TFs was achieved through STEM analysis on 26,969 genes (FPKM > 5 in a single sample). The analysis revealed that 7 out of the 30 profiles were significantly clustered: Profile 14, Profile 18, Profile 26, Profile 5, Profile 12, Profile 4, and Profile 22 (Figure 7A and Figure S5). These significant clusters comprised 6636, 2803, 1323, 1274, 984, 718, and 660 genes, respectively (Figure S6). Subsequently, we examined the expression levels of *MYB* and *NAC* genes, as well as the genes involved in lignin, cellulose, and hemicellulose biosynthesis, with FPKM values greater than 10 within these significant profiles. The results showed that the key genes related to cellulose and hemicellulose biosynthesis were predominantly clustered within Profile 14, with highly expressed genes (FPKM > 100) concentrated in this profile (Figure 7B–H). Among the 11 potential key genes for cellulose and hemicellulose biosynthesis identified by RT-qPCR, 10 were assigned to the Profile 14 (Figure 4 and Figure 7B). Additionally, lignin biosynthesis genes with high expression levels (FPKM > 100) were found across Profile 14, Profile 18, Profile 22, Profile 26, and Profile 4, with a majority located within Profile 14 (Figure 7B–H). Out of the 10 key lignin biosynthesis genes validated by RT-qPCR, 7 were categorized within Profile 14 (Figure 2 and Figure 7B).

Among the 13 validated *NAC* and *MYB* genes, 2 were located in Profile 4, 3 in Profile 5, 1 in Profile 12, and 1 in Profile 14 (Figure 7B,C,G,H). The three TF genes within Profile 5, PITA_17809 (*NAC*), PITA_39801 (*MYB*), and PITA_15281 (*MYB*), were predominantly expressed in the stem xylem, while PITA_24173 (*NAC*) in Profile 14 showed a similar expression pattern (Figure 6). The genes within Profile 4 were anticipated to be highly expressed in the bark and root samples (Figure 7A), with the RT-qPCR results confirming that PITA_04201 (*NAC*) was highly expressed in the stem bark, and PITA_18689 (*MYB*) was predominantly expressed in the root (Figure 6). The genes within Profile 12 were expected to exhibit low expression in the needle and root samples, which was corroborated by RT-qPCR, showing that PITA_18754 (*MYB*) exhibited low expression only in the root (Figure 6). These observations suggest that there may be minor discrepancies between the expression patterns obtained from the transcriptomic data and those derived from the RT-qPCR analysis, possibly due to temporal and spatial differences in the samples used for each method. Importantly, the seven identified MYB and NAC TFs do not belong to the classical MYB subgroups or VNS clades associated with SCW formation but rather belong to alternative evolutionary branches (Figure 5C,D). This indicates that the seven NAC and six MYB TFs identified in this study provide a significant extension to the previously characterized MYB and NAC TFs implicated in SCW formation in *P. taeda*.

## 3. Discussion

### 3.1. Lignin, Cellulose, and Hemicellulose Biosynthesis in P. taeda: A Complex and Multifaceted Process

The biosynthesis of lignin, cellulose, and hemicellulose in *P. taeda*, as in other species, is a complex process involving multiple pathways and the intricate coordination of various enzymatic activities [5,6,10,11,12,15,16,21,22,23,24,25]. Notably, the expression levels of genes encoding these enzymes significantly influence the biosynthesis of lignin, cellulose, and hemicellulose. For instance, silencing *IRX9L*, *IRX10L*, and *IRX14* disrupts xylan biosynthesis, while the overexpression of *RWA* enhances xylan acetylation in *Populus* [20,24]. Similarly, the downregulation of *CAD* reduces lignin content in *Paspalum notatum*, and in *A. thaliana*, the *ccd4 ccd5* double mutant exhibits lower lignin accumulation compared to the wild type (WT) [45,46]. However, the specific enzyme-encoding genes involved in the biosynthesis of lignin, cellulose, and hemicellulose in *P. taeda* remain unidentified.

Transcriptome-based gene identification is a widely adopted approach for studying genes involved in lignin, cellulose, and hemicellulose biosynthesis. For example, Zhang et al. identified 20 lignin biosynthesis-related genes during the pigmentation process in *Ziziphus jujube* [47], and Chen et al. identified 42 lignin-related genes in *Styphnolobium japonicum* [48]. In the species of the genus *Pinus*, a larger number of genes associated with the biosynthesis of lignin, cellulose, and hemicellulose have been reported. Ni et al. identified 122 lignin-related genes, 37 cellulose-related genes, and 71 hemicellulose-related genes in *P. massoniana* [49]. Similarly, Kim et al. identified 56 genes involved in lignin biosynthesis and 64 genes related to cellulose and hemicellulose biosynthesis in *P. densiflora* [10]. Furthermore, Nguyen et al. reported the identification of 34 key genes associated with lignin biosynthesis and 36 genes involved in the biosynthesis of cellulose, galactoglucomannan, and xylan in *P. densiflora* [50]. In this study, we identified 188 genes related to lignin biosynthesis and 78 genes involved in cellulose and hemicellulose biosynthesis (Figures S2 and S3). It is noteworthy that these genes were identified through transcriptomic data from bark, bud, needle, root, and twig tissues, rather than a comprehensive whole-genome analysis. Previous studies have shown that sequencing depth, sample size, and tissue developmental stage significantly impact the number of genes identified via transcriptomic analysis [51,52]. Thus, the 266 genes identified in this study likely represent only a subset of the total genes involved in lignin, cellulose, and hemicellulose biosynthesis in *P. taeda*. To uncover additional genes, increasing sample size, improving sequencing depth, or utilizing whole-genome data would be necessary.

### 3.2. The Crucial Role of Non-Classic SCW-Related NAC and MYB TFs in P. taeda

NAC and MYB TFs are recognized as the master regulators of SCW formation. VNS TFs, a subgroup of the NAC family, act as primary regulators in SCW formation, highlighting their crucial role in this process [26]. Previous studies have identified five VNS TFs in the *P. taeda*, with PtaVNS1, PtaVNS2, PtaVNS3, and PtaVNS5 promoting SCW formation, albeit with varying efficiency—PtaVNS3 being the least efficient [37]. Interestingly, our analysis revealed that in addition to VNS TFs, non-VNS NAC TFs also play significant roles in SCW formation in *P. taeda*. Similar findings have been reported in studies of *Eucalyptus grandis*, *A. thaliana*, and *Populus*. For example, EgNAC141, which positively regulates lignin biosynthesis, does not belong to the VNS subfamily [53]. Likewise, XYLEM NAC DOMAIN 1 (XND1), a non-VNS subfamily TF, acts as a negative regulator of wood formation in *A. thaliana* and its orthologs in *Populus* [54,55]. Notably, PITA_04201, a NAC gene in *P. taeda*, exhibited an expression pattern similar to PtaVNS2 and PtaVNS5, suggesting its involvement in SCW formation (Figure 6). These findings indicate that non-canonical NAC genes also play significant roles in SCW biosynthesis in *P. taeda*.

Similarly, MYB TFs, which serve as the secondary regulators of SCW formation, are indispensable to this process [26]. Most MYB TFs involved in SCW formation belong to specific evolutionary clades, including MYB4/7/32, MYB58/63, MYB42/85, MYB103, MYB46/83, MYB55/61, and SWAM [56]. The roles of MYB TFs from these clades in wood formation have been extensively studied. For instance, PtrMYB24 (MYB46/83 subclade) positively regulates lignin and cellulose biosynthesis in pear [57], and PlMYB43 (MYB42/85 clade), PlMYB83 (MYB46/83 clade), and PlMYB103 (MYB103 clade) activate lignin biosynthesis in *Paeonia lactiflora* [58]. Additionally, PtrMYB3 and PtrMYB20 (MYB46/83 clade) activate the biosynthesis of cellulose, xylan, and lignin in *P. trichocarpa* [59]. However, the roles of MYBs outside of these clades in SCW formation should not be overlooked. Previous research has implicated PtaMYB1, PtaMYB4, PtaMYB8, and PtaMYB14 in SCW regulation in *P. taeda*, corresponding to the MYB42/85, MYB46/83, MYB55/61, and MYB4/7/32 clades, respectively [9,38,39,40,41,42,43]. In contrast, among the six key MYB TFs identified in this study, only PITA_17636 and PITA_12548 belong to the same clade as PtaMYB14, while the other four MYB TFs are not classified into canonical SCW-related MYB clades. Although these MYB TFs may contribute to SCW formation, further validation is required.

## 4. Materials and Methods

### 4.1. Transcriptome Data Analysis

The raw data from the barks, roots, needles, buds, and twigs of loblolly pine were acquired from the NCBI database (SRP304195) [44]. The Fastp v0.23.2 software was employed to process the raw data [60]. After eliminating adaptor sequences, low-quality reads (single base error rate higher than Q20), reads shorter than 150 bp, and reads containing poly(N), we obtained the cleaned data. The genome data of loblolly pine were downloaded from the National Center for Biotechnology Information (NCBI) database (https://www.ncbi.nlm.nih.gov/datasets/genome/?taxon=3352, accessed on 20 April 2024) [61]. The Hisat2 v2.2.0 software was then utilized to construct the loblolly pine genome index and align the cleaned data to the genome [62]. Based on the alignment outcomes, the Stringtie v2.2.3 software was used to assemble the RNA-seq reads into transcripts [63]. Read counts were calculated using the FeatureCounts software (included in the subread v2.0.6 software package) [64]. The calculated read counts were converted into fragments per kilobase of exon model per million mapped fragments (FPKM) values to quantify gene expression levels.

The open reading frame (ORF) of each transcript was predicted using the Complete ORF Predict module of the TBtools v2.124 software 54. The transcript with the longest ORF was selected as the representative transcript of each gene. The representative ORF sequence of each gene was translated into a protein sequence using the Batch Translate CDS to Protein module of the TBtools v2.124 software [65]. The protein sequences were used for the identification of proteins related to lignin, cellulose, and hemicellulose biosynthesis, TF prediction, and phylogenetic tree construction.

### 4.2. Identification of Lignin, Cellulose, and Hemicellulose Biosynthesis-Related Proteins

According to information provided by the Kyoto Encyclopedia of Genes and Genomes (KEGG) database (https://www.kegg.jp/, accessed on 22 April 2024), we identified the enzyme commission (EC) numbers of enzymes involved in lignin, cellulose, and hemicellulose biosynthesis. Based on the EC numbers, we downloaded the enzyme protein sequences from the UniProt database (https://www.uniprot.org/, accessed on 22 April 2024). The retrieved protein sequences (reference protein sequences) were used to identify homologous proteins in loblolly pine by performing a local BLAST. The homologous proteins in loblolly pine were required to have more than 50% identity with the reference protein sequences.

### 4.3. Prediction of TFs

The Plant TF Database (https://planttfdb.gao-lab.org/index.php, accessed on 25 April 2024) was utilized to identify TFs within the representative protein sequences obtained from the RNA-seq analysis.

### 4.4. Plant Materials, Total RNA Extraction, and cDNA Synthesis

To identify the genes associated with SCW formation, samples of stem bark, stem xylem, mature needles, and roots were collected to assess the expression levels of potential SCW-related genes (Figure S7). These samples were taken from two-year-old loblolly pines cultivated at the nursery of Jiangxi Academy of Forestry in China. Each sample type consisted of three biological replicates. The collected tissues were immediately frozen in liquid nitrogen and stored in a −80 °C ultra-low temperature freezer. Total RNA was extracted using the RNAprep Pure Plant Plus Kit (Tiangen, Beijing, China), and the quality and quantity of the RNA were assessed using 2% agarose gel electrophoresis and an Agilent 2100 Bioanalyzer (Agilent Technologies, Santa Clara, CA, USA). cDNA was synthesized using the PrimeScriptTM FAST RT reagent Kit with gDNA Eraser (Takara, Beijing, China).

### 4.5. RT-qPCR Analysis of TF Genes and Genes Involved in Lignin, Cellulose, and Hemicellulose Biosynthesis

Fifteen genes involved in lignin biosynthesis, thirteen genes related to cellulose and hemicellulose biosynthesis, and twenty-one TF genes were selected for RT-qPCR analysis. EF1α was used as the reference gene. Primers for *EF1*α, *PtaVNS1*, *PtaVNS2*, *PtaVNS4*, and *PtaVNS5* were obtained from a previous study [37]. All the primer sequences are listed in Table S1. RT-qPCR was performed using the TB Green^®^ Premix Ex Taq™ II kit on the CFX96 Real-Time PCR Detection System (Bio-Rad, Hercules, CA, USA). Detailed RT-qPCR parameters were set according to the kit instructions. The relative expression levels of the genes were calculated using the 2^−ΔΔCt^ method.

### 4.6. Construction of the Phylogenetic Trees of MYB and NAC TFs

To construct the phylogenetic trees of MYB and NAC TFs, we downloaded the MYB and NAC protein sequences of *A. thaliana* from the Plant TF Database. Ten loblolly pine MYB proteins and twelve *A. thaliana* MYB proteins were used to construct the MYB protein phylogenetic tree, and eleven loblolly pine NAC proteins and sixteen *A. thaliana* NAC proteins were used to construct the NAC protein phylogenetic tree. The MEGA X software was applied to construct a Neighbor-Joining tree with the Jones–Taylor–Thornton model and 1000 bootstrap replications [66].

### 4.7. STEM Analysis of Filtered Genes

To identify genes with specific expression patterns that may be associated with SCW formation, we performed a STEM analysis to cluster genes into different expression profiles based on their expression levels (FPKM value) in barks, roots, needles, buds, and twigs. The genes with FPKM > 5 in a single sample were input into the OmicStudio platform (Bioinformatic analysis was performed using the OmicStudio tools at https://www.omicstudio.cn/tool, accessed on 28 April 2024) to perform the STEM analysis. For data normalization, the log normalization method was employed, while the STEM clustering method was used for clustering. The analysis was set to identify 30 expression profiles, with a unit change of 1. The highly expressed genes in the significantly clustered profiles were considered as the focus of the research.

## 5. Conclusions

As an economically significant timber species, understanding the molecular mechanisms underlying SCW formation in *P. taeda* is of considerable importance. In this study, we identified 188 lignin biosynthesis-related genes and 78 genes involved in the biosynthesis of cellulose and hemicellulose, marking the first systematic identification of such genes in *P. taeda*. From this set of 266 genes, 28 key synthase genes were selected for further RT-qPCR analysis, revealing their high expression in the stem bark and stem xylem tissues. Moreover, the STEM analysis indicated that genes with high expression related to lignin, cellulose, and hemicellulose biosynthesis were predominantly grouped within Profile 14, underscoring the critical importance of these genes in SCW formation in *P. taeda*. Additionally, we identified seven key NAC and six key MYB TFs, with the RT-qPCR results demonstrating that most of these key *NAC* and *MYB* genes exhibited expression patterns distinct from those of classical SCW-related NAC and MYB genes. These findings represent a substantial contribution to the understanding of SCW formation in *P. taeda*, providing a valuable genetic foundation for future studies on the regulatory mechanisms of SCW formation.

## Data Availability

Data is contained within the article and Supplementary Materials.

## Supplementary Materials

The following supporting information can be downloaded at https://www.mdpi.com/article/10.3390/ijms252111805/s1.
