# Supplementary material for: Unveiling Key Genes and Unique Transcription Factors Involved in Secondary Cell Wall Formation in Pinus taeda
Source: Int J Mol Sci. 2024 Nov 3;25(21):11805. doi: 10.3390/ijms252111805 (PMC11545933; doi:10.3390/ijms252111805)
Supplement: Supplementary file 1 [file ijms-25-11805-s001.zip › ijms-3224991-supplementary.pdf]

Table S1 Primers for RT-qPCR analysis

Name	Primer sequence (5'-3')	Name	Primer sequence (5'-3')
PITA_02510-F	CGTGACTCCTCTCTTAATAC	PITA_18754-R	ACCAAGCCGAATGTAGTA
PITA_02510-R	CGATGATACCTTCTTCTCTG	PITA_24173-F	CCGTCTTGCTGATGTTAG
PITA_20438-F	TTCCTATTCATTGCTCTCTG	PITA_24173-R	AGTTGAGGAGGGTTGATT
PITA_20438-R	CCATCCGTTTCTCATTGT	PITA_17809-F	TGGAACAGAAGGACAGAT
novel.14125-F	CAGAGATAGCCTAAAGTTCG	PITA_17809-R	GAAGAGGATGAGACACAAC
novel.14125-R	ATGAGTTACAGTGACCAATG	PtaVNS1-RT-F	GGCTTGAGACGAACGAGAAC
PITA_14161-F	CACGATTCCTTTCCTCAC	PtaVNS1-RT-R	TGTTTGCAGCCTGAGAATTG
PITA_14161-R	GGTCTTGTCCTCACTCTT	PtaVNS2-RT-F	CCCATACCTGATATGACTGTGCTTC
PITA_41378-F	GACAGGCAGTTTCCATTT	PtaVNS2-RT-R	TCATTTTCCTGCAAAGCTCCAGAG
PITA_41378-R	AACCACTAACAGAGCAATC	PtaVNS3-RT-F	TGTAAGCGAATTGGCATGAG
PITA_29315-F	CAACGGAATCAAGAAGGT	PtaVNS3-RT-R	TCTTTGGCGTCTTTGTTGTG
PITA_29315-R	GAATGAAGAGGCAGATGG	PtaVNS5-RT-F	CTCCTAATTCTTCTTCCGATCATCA
PITA_44880-F	TCGGATTGCCTATTATTGAG	PtaVNS5-RT-R	ATCAATGAGCAGTCATGGAATTTGT
PITA_44880-R	GACGCTTGTGGTAGTTTA	Pta-EF1α-F	TACAAAATTGGTGGTATTGGGACTG
PITA_30025-F	TGGATGATGTAGACTTGGT	Pta-EF1α-R	CATGGGTTTGGTTGGTATCATCTTA
PITA_30025-R	CCTCTGCTTGAAACTTGT	PtaMYB4RT-F	AGATGTATTGGCGTTAAGAC
PITA_28257-F	AAGCACAGGAAGAGATAGA	PtaMYB4RT-R	TGGGAAATGAGGTTGTTG
PITA_28257-R	CAGGTATGGTAGATGAGGA	PtaMYB8RT-F	CTGCCTTTCTTCTTTGCTA
PITA_03866-F	CAAGGTCAAGGAAGATGG	PtaMYB8RT-R	TTATCGGTATTGTCCCAGTA
PITA_03866-R	CATTGTTTCGCTGGTGTA	PtaMYB1RT-F	TTACTGGAACACGAGGAT
PITA_14168-F	CTGAGATAGCGAAGCAAT	PtaMYB1RT-R	ATTGCTGCTCTGATTCTG
PITA_14168-R	AGATGAGATGAGCACAATG	PtaMYB14RT-F	GGGAACAAATGGTCTCTTAT
PITA_31897-F	GCGAAGATTGTGAATGAAG	PtaMYB14RT-R	AGAACGGTGTAGGTAGTG
PITA_31897-R	TTGCTATGATTTGGAGGAC	PITA_09044-F	GCCTAACATTTCTGGACTT
novel.8888-F	AGTGACTTTGCTGAGATG	PITA_09044-R	GCGTGGATATTGACTCTG
novel.8888-R	GACTCTGAACTCGTAACAA	PITA_04408-F	GAACGGAGAGCAATGAAG
PITA_21162-F	AGAATGGTGGAACGATAAC	PITA_04408-R	ACTGTGACCTAAGAATACCT
PITA_21162-R	ATTAACGATACGCAGAAGG	PITA_07279-F	GAAGAGTGTTGCTGAGAAT
PITA_12328-F	GAAGAAATCGCTGGTGTT	PITA_07279-R	TCGTTGAAGAGTATGGAATC
PITA_12328-R	CGAATGATTGAGTCTCTGAA	PITA_05742-F	GCAGCCTCATCAACTATT
PITA_04201-F	TTCTTCTGTCCTCGTGAT	PITA_05742-R	ACCTCAACACTTCTCCAA
PITA_04201-R	CAATCTGTTCGTTCTCCAT	novel.12743-F	AGGATACTGGTGATACTCG
PITA_29872-F	AACACCATCCTCTTCTCA	novel.12743-R	CGCTGACTTGCTTGATAG
PITA_29872-R	GCACATTCCATTCAACATC	PITA_07011-F	ACCACAGATTAGCACATTC
PITA_39801-F	ATGGAATGGATGAACTCTG	PITA_07011-R	TCATTCCTCCACCATTCA
PITA_39801V	CTCTGTGTTGTTGTTGTTG	novel.13077-F	GAAGTTGCTATGATGAAGGA
PITA_17325-F	GAAGAGGCAATGGAACAA	novel.13077-R	TGCGAGGACCATAAGTAT
PITA_17325-R	ATGGAAGTAATGGCAGAAG	PITA_34043-F	TCCACTACCAAGAAGAGAA
PITA_17636-F	TACTTGTCTATGGAGGGAAA	PITA_34043-R	GTATCGCTGTCCAAATGT
PITA_17636-R	ATTGGCTCTGATTCTGTATG	PITA_47988-F	GGTGTGGTTATTCTTGATGA
PITA_18689-F	GAGCGTAGAGCAGAATAAG	PITA_47988-R	CTGCTTCTTTGTGGCTAT
PITA_18689-R	GTCACATCCTCAACAAGAA	PITA_39609-F	CTACCGATGGCAGATACT

PITA_12548-F	GCCTAATGTTGTTGAAGAAG	PITA_39609-R	GCAGAAGAAGATGATGAAGA
PITA_12548-R	GTTACTCCTGCCAAATGAT	novel.14875-F	CGCCTTCTCCATAATACAAT
PITA_15281-F	TGCGAATCTACAGGAAAC	novel.14875-R	TTACAATCCTGCCATCTTAG
PITA_15281-R	GTGCTATTGTTGTGAGAGT	PITA_42319-F	CGAATGTGCCTTGATGAT
PITA_10236-F	TTAGAGGGCATTCAGAGG	PITA_42319-R	GGAAACGAACTGGAAACT
PITA_10236-R	TTGTTCATCACCACTTGTAG	PITA_39478-F	GGACATAGAAGCAGTAGAAG
PITA_18754-F	GAACAGGAGAGAAAGAAGG	PITA_39478-R	AGAAGAGTAGAGGCAACA

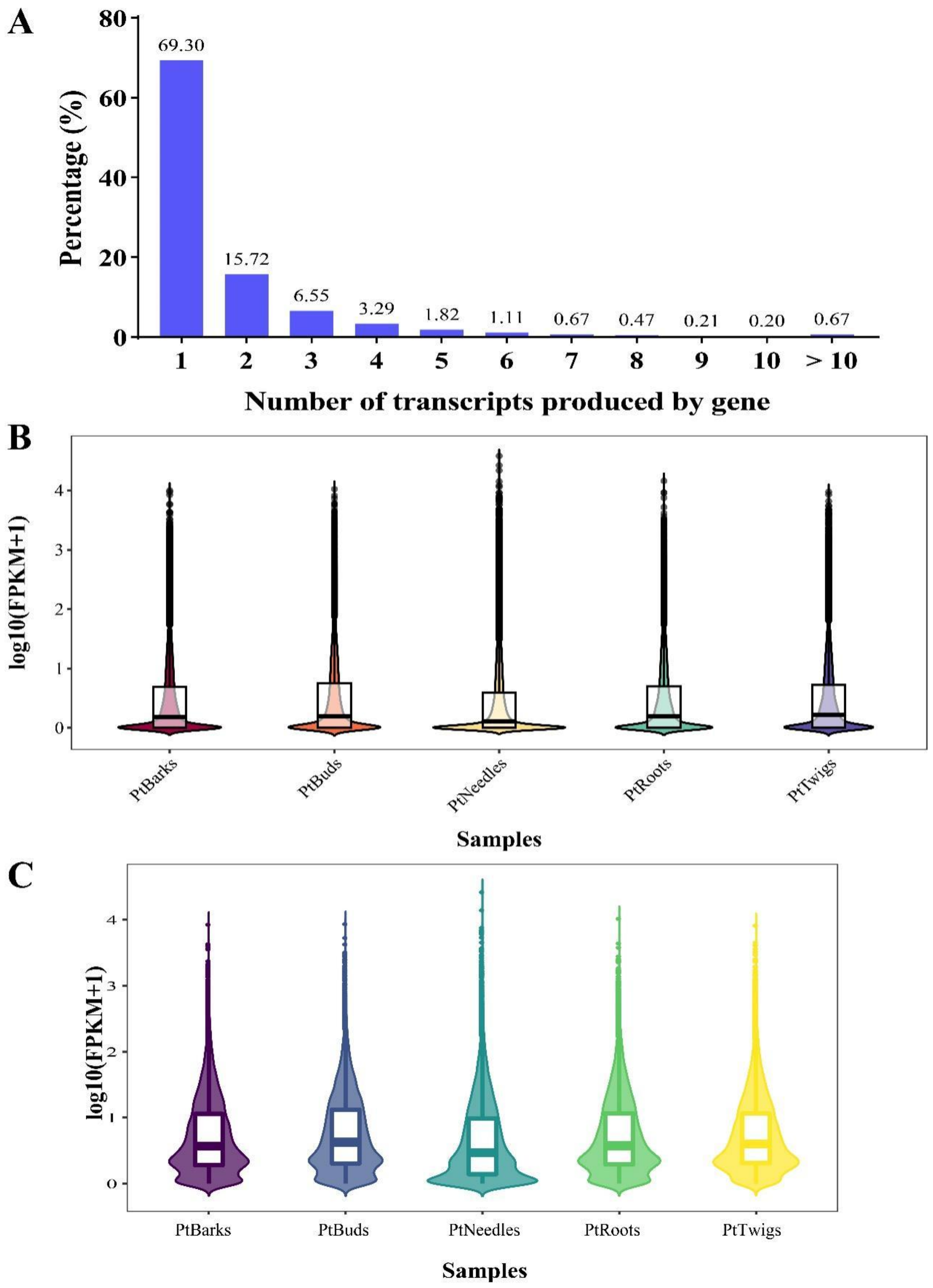

Figure S1 Overview of RNA sequencing. (A) Statistics of the transcripts produced by each gene. (B) Violin plot showing gene expression levels across different samples.(C) Violin plot of gene expression levels for genes with FPKM values greater than 1 in at least one sample.

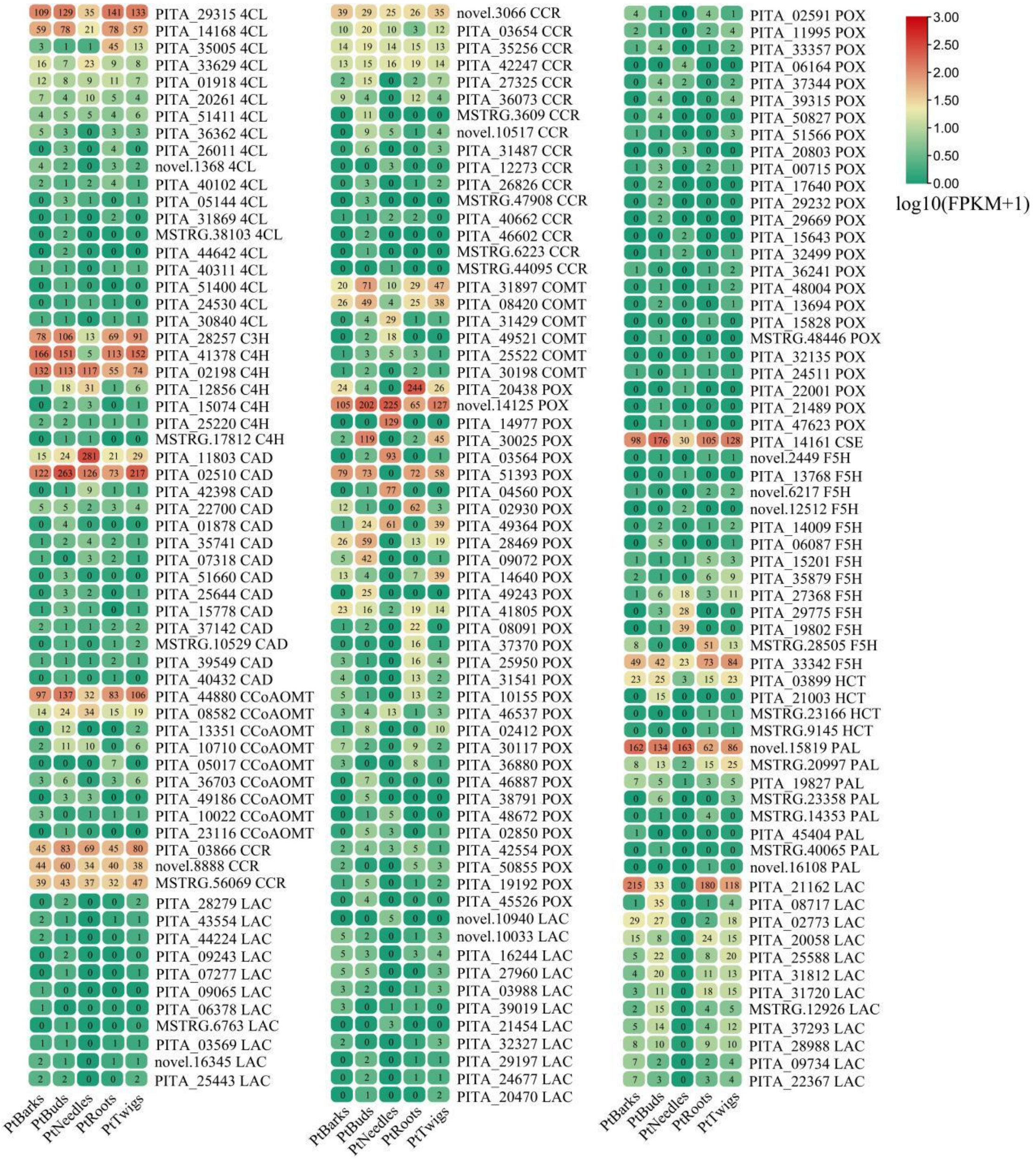

Figure S2 Heatmap of the 188 lignin biosynthesis-related genes.

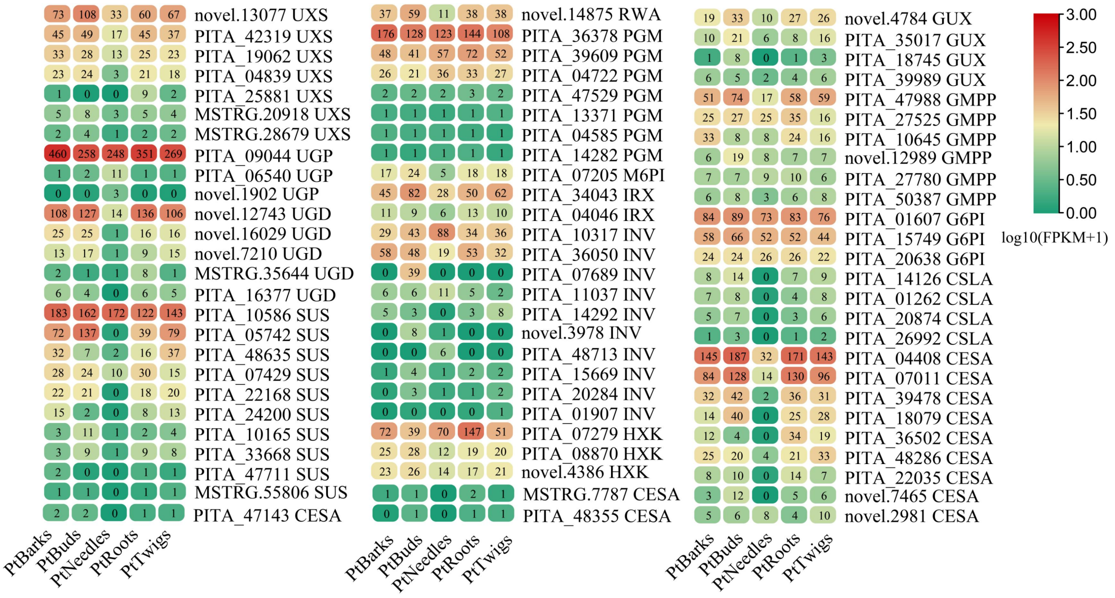

Figure S3 Heatmap of the 78 genes related to cellulose, hemicellulose (xylan), and galactoglucomannan biosynthesis.

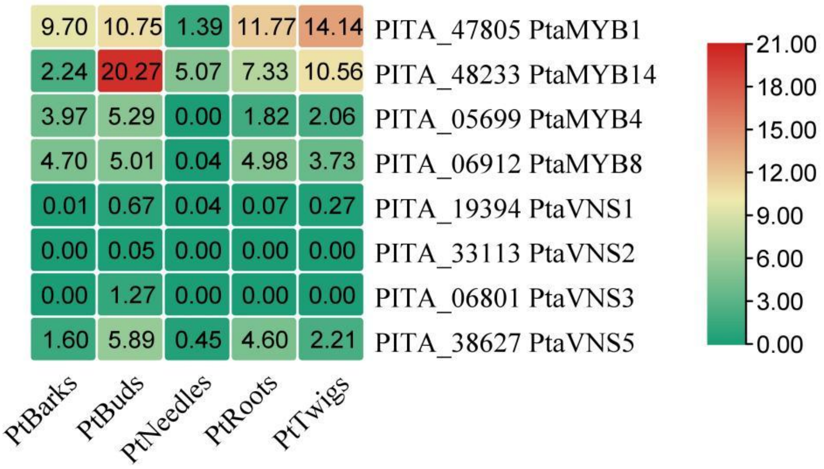

Figure S4 Heatmap of the reported MYB and NAC genes. The numerical values in the heatmap represent the FPKM values of genes.

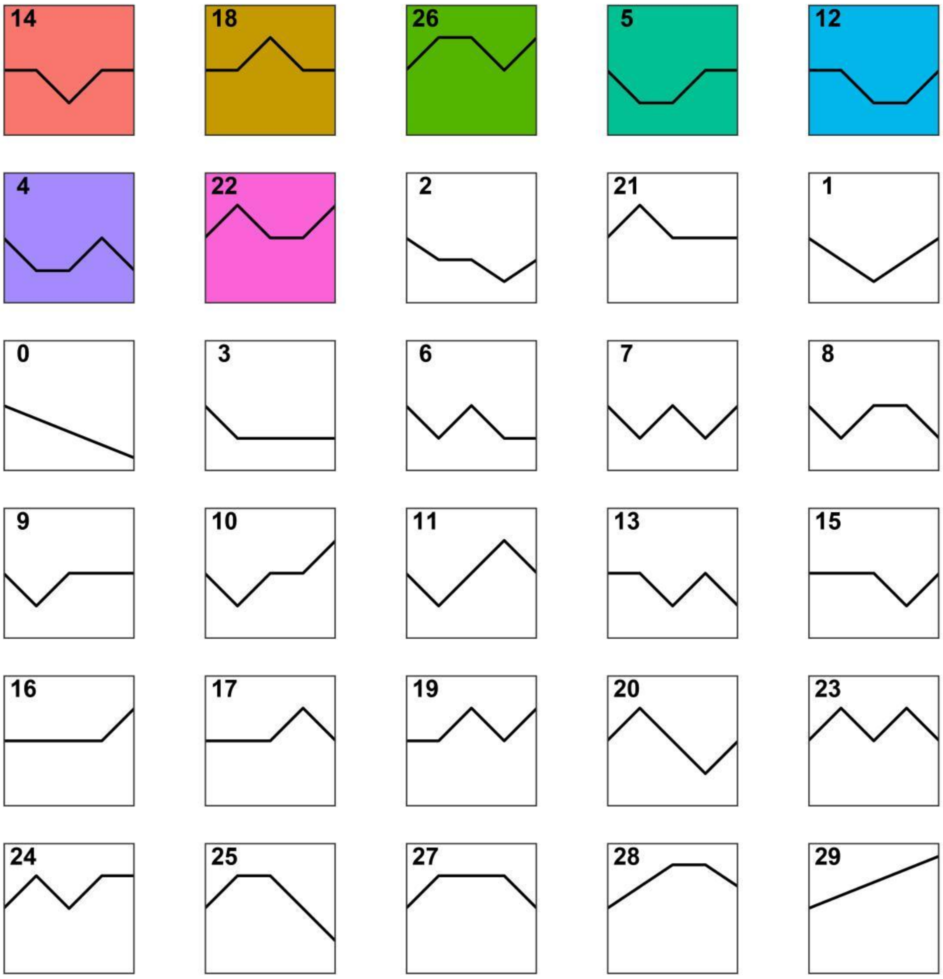

Figure S5 All profiles from STEM analysis results.

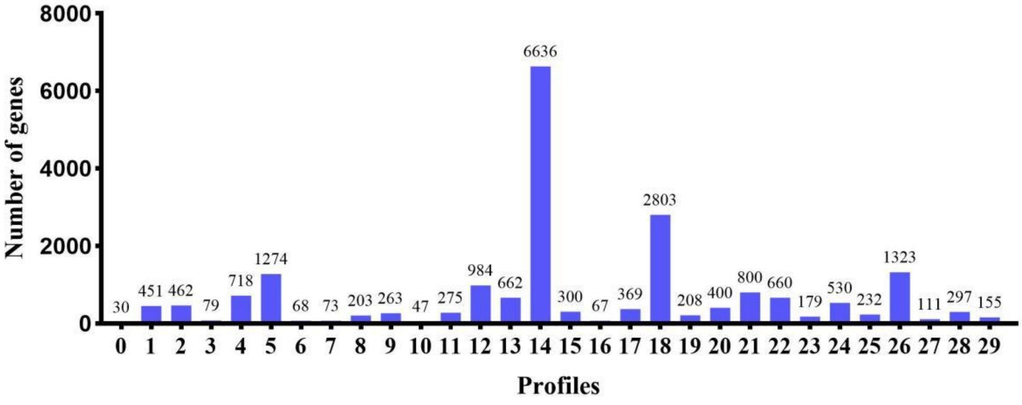

Figure S6 Statistics of gene numbers in 30 profiles.

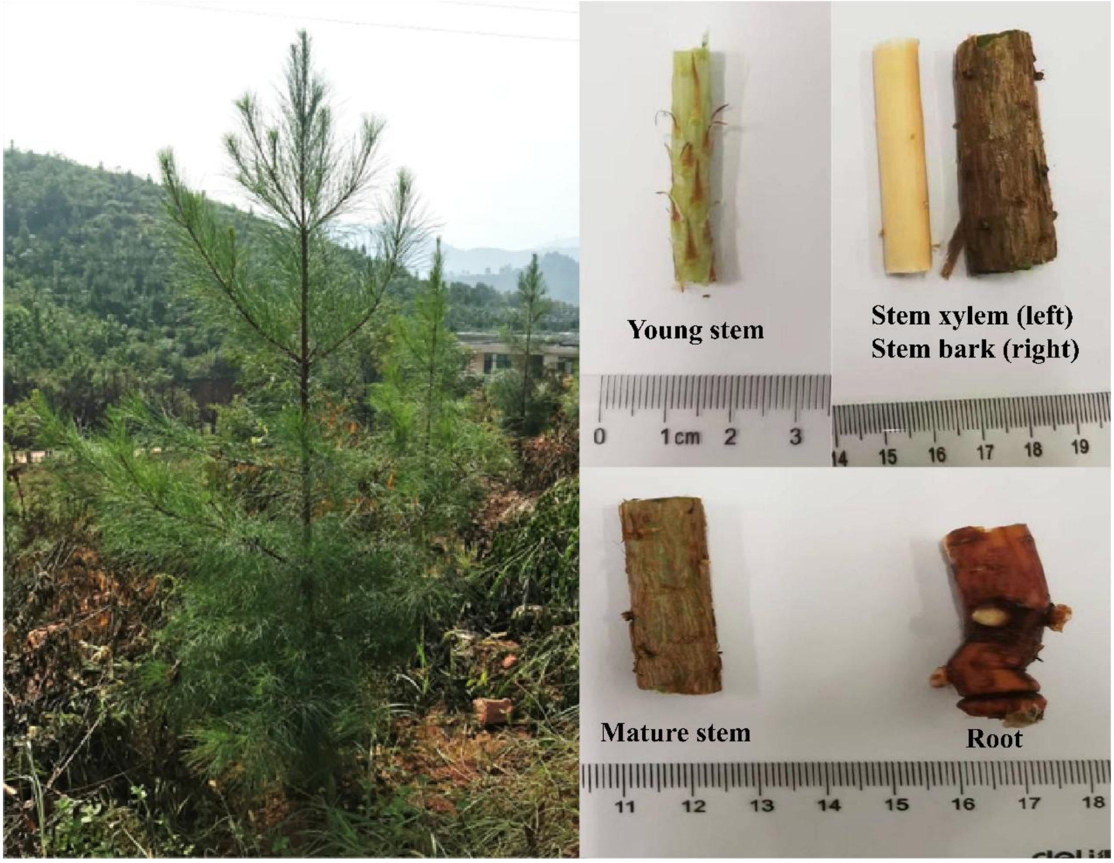

Figure S7 Plant materials used for RT-qPCR analysis
